# Associations Between Device-Measured Physical Activity and Glycemic Control and Variability Indices Under Free-Living Conditions

**DOI:** 10.1089/dia.2021.0294

**Published:** 2022-03-14

**Authors:** Douae El Fatouhi, Harris Héritier, Chloé Allémann, Laurent Malisoux, Nasser Laouali, Jean-Pierre Riveline, Marcel Salathé, Guy Fagherazzi

**Affiliations:** ^1^ “Exposome, Heredity, Cancer, and Health” Team, Center of Research in Epidemiology and Population Health (CESP), Inserm U1018, Paris-Saclay University, UVSQ, Gustave Roussy, Espace Maurice Tubiana, Villejuif, France.; ^2^ Digital Epidemiology Laboratory, School of Life Sciences, École Polytechnique Fédérale de Lausanne (EPFL), Geneva, Switzerland.; ^3^ Physical Activity, Sport and Health Research Unit, Department of Population Health, Luxembourg Institute of Health (LIH), Strassen, Luxembourg.; ^4^ Department of Biostatistics and Epidemiology, School of Public Health and Health Sciences, University of Massachusetts, Amherst, Massachusetts, USA.; ^5^ Department of Diabetes and Endocrinology, Assistance Publique-Hôpitaux de Paris, Université de Paris, Lariboisière Hospital, Paris, France.; ^6^ Inserm U1138, Immunity and Metabolism in Diabetes (ImMeDiab Team), Centre de Recherches des Cordeliers, Paris, France.; ^7^ Deep Digital Phenotyping Research Unit, Department of Population Health, Luxembourg Institute of Health (LIH), Strassen, Luxembourg.

**Keywords:** Continuous glucose monitoring, Physical activity, Step count, Wearable activity trackers, Free-living, Digital health

## Abstract

**Background:**

Disturbances of glycemic control and large glycemic variability have been associated with increased risk of type 2 diabetes in the general population as well as complications in people with diabetes. Long-term health benefits of physical activity are well documented but less is known about the timing of potential short-term effects on glycemic control and variability in free-living conditions.

**Materials and Methods:**

We analyzed data from 85 participants without diabetes from the Food & You digital cohort. During a 2-week follow-up, device-based daily step count was studied in relationship to glycemic control and variability indices using generalized estimating equations. Glycemic indices, evaluated using flash glucose monitoring devices (FreeStyle Libre), included minimum, maximum, mean, standard deviation, and coefficient of variation of daily glucose values, the glucose management indicator, and the approximate area under the sensor glucose curve.

**Results:**

We observed that every 1000 steps/day increase in daily step count was associated with a 0.3588 mg/dL (95% confidence interval [CI]: −0.6931 to −0.0245), a 0.0917 mg/dL (95% CI: −0.1793 to −0.0042), and a 0.0022% (95% CI: −0.0043 to −0.0001) decrease in the maximum glucose values, mean glucose, and in the glucose management indicator of the following day, respectively. We did not find any association between daily step count and glycemic indices from the same day.

**Conclusions:**

Increasing physical activity level was linked to blunted glycemic excursions during the next day. Because health-related benefits of physical activity can be long to observe, such short-term physiological benefits could serve as personalized feedback to motivate individuals to engage in healthy behaviors.

## Introduction

Glycemic control can be defined as the ability to maintain glycemia or blood glucose within the recommended target range (70–180 mg/dL or 3.9–10 mmol/L).^1^ In healthy people, blood glucose levels are strictly regulated.^2,3^ Disturbances of glycemic control are generally detected in the general population using different tools such as fasting plasma glucose, 2 h postload plasma glucose, and HbA1c. Elevated levels of these metrics have been associated with increased risk for type 2 diabetes,^4–6^ cancer,^7–10^ and cardiovascular events^11–14^ among individuals without diabetes.

Glycemic variability represents the fluctuations between high and low blood glucose levels.^15^ However, it is a broad term that can include many concepts and that can be assessed in very different ways.^11,15–17^ The first concept refers to the day-to-day or visit-to-visit variability over months to years of fasting plasma glucose or HbA1c evaluated using standard deviation (SD) or coefficient of variation (CV) of repeated measures. The second meaning relates to postprandial glucose excursions. Finally, the last applies to the intraday or short-term glycemic variability using continuous glucose monitoring (CGM) systems, and for which a wide range of indices have been proposed to characterize it. Some studies revealed associations between large visit-to-visit glycemic variability and increased risks of all cancers^17^ and cardiovascular events^11,15,18^ among populations without diabetes as well as complications in people with diabetes.^15,17^ Moreover, Jang et al. showed that individuals without diabetes with large glycemic variability tended to have increased HbA1c, fasting plasma glucose, postprandial glycemia, and insulin resistance, which are all important risk factors for type 2 diabetes.^11^

Therefore, it is key to identify lifestyle-related drivers to reduce glycemic variability and improve glycemic control. This may contribute to an improvement of cardiometabolic health in the general population and, in individuals with diabetes, an improved quality of life and a decreased risk of diabetes-related complications. It has been established that physical activity was associated with a lower risk of type 2 diabetes and with improved glycemic control markers.^19–21^ A recently published systematic review, including 10 randomized controlled trials, revealed limited evidence showing that physical activity could reduce CGM-based measures of glycemic variability in people with type 2 diabetes.^22^ Long-term health benefits of physical activity are well documented but less is known about the timing of physical activity's potential short-term effects on glycemic control and variability in free-living conditions.

In the past few years, mHealth technologies, such as smartphone health apps or consumer wearable activity trackers that allow the monitoring of health parameters and behaviors, have become widely available. Their improvements by manufacturers and use are on a constant rise.^23,24^ Simultaneously, there have been important developments of CGM devices, which have made them more affordable and easily accessible.^25^ Besides, mHealth technologies and CGM systems can also give researchers access to more accurate, objective, and continuous measurements, which opens new research perspectives, in particular, to better understand the short-term interrelations between physical activity and glycemic control and variability.

The aim of this study was to explore the associations of device-measured physical activity expressed as daily step count with various glycemic control and variability indices derived from 13 days of CGM in adults without diabetes from the Food & You (FAY) digital cohort.

## Materials and Methods

### Study population

Data used for this study were collected as part of the FAY study, which is a digital cohort that aims to use dietary intake, physical activity, sleep, microbiome, and glucose data to build a model that predicts individuals' postprandial glucose responses. The FAY study is a citizen science research project that also aims to increase scientific knowledge, raise general awareness of scientific questions, and promote exchange between scientists and citizens. The study is conducted by the Digital Epidemiology Lab located in Geneva and is sponsored by the Swiss Federal Institute of Technology Lausanne (*École Polytechnique Fédérale de Lausanne, EPFL*). The recruitment started in February 2018 and is still ongoing. All participants gave their informed consent, and the study was authorized by the Geneva Ethics Commission (*Commission Cantonale d'éthique de la recherche Genève; trial identification number 2017-02124*).

FAY study inclusion criteria required participants to be at least 18 years of age, to have a postal address in Switzerland, to own a smartphone (minimum version iOS 9/Android 5), to have a good understanding of French, German, or English, and to provide an informed consent. Study exclusion criteria included the following: pregnancy, breastfeeding, being on dialysis, having been diagnosed or prediagnosed type 1 or type 2 diabetes mellitus, having used antibiotics in the 3 months before enrollment, using chronic immunosuppressive medication, being critically ill, suffering from a chronic gastrointestinal disorder (inflammatory bowel disease, celiac disease) or from any active neuropsychiatric disorder, having suffered from chronically active inflammatory or neoplastic disease in the 3 years before enrollment, and having suffered from myocardial infarction or a cerebrovascular accident in the 6 months before enrollment.

### Study design and measurements

The FAY study is characterized by two tracking weeks during which each participant wore a CGM device. During these two tracking weeks, participants were instructed to take photos of all their meals and food intakes using the MyFoodRepo smartphone app, which is available on App Store, Google Play Store, and other Android app stores.^26^ This app can be downloaded in smartphones with at least the iOS 9 version or the Android 5 version and can only be used by participants of the FAY study or other partner cohorts.

FAY study participants were also asked to log their daily physical activities and sleep duration into the smartphone-adjusted website that was developed for this digital cohort. Participants were asked to report the start time, end time and the intensities (light, moderate, or intense) of their daily physical activities. Participants who use mHealth technologies such as smartphone health apps or consumer-based wearable activity trackers to monitor their physical behaviors (physical activity and sleep) in their daily lives were given the option to securely share, with the study investigators, their device-based 2-week data extracted from their personal account of their device's brand, instead of having to fill the daily online questionnaires.

Before receiving the CGM system, participants had to answer different online questionnaires. Detailed information about health conditions, medical background, treatments, lifestyle, diet, anthropometric measures (e.g., height, weight, hip, and waist circumferences), educational level, and income were thus recorded. Participants were also requested to provide a single stool sample during the tracking weeks. More details on the study and study methodology can be found elsewhere.^27^ A description of the study can also be found on the ClinicalTrial.gov platform (number NCT03848299).

### Physical activity assessment

The present study focuses on FAY study participants that used mHealth technologies to follow their physical behaviors during the 2-week tracking period and who agreed to share their personal data.

Different types of mHealth technologies and brands were used across the FAY study participants such as Fitbit and Garmin watches, Apple devices (iPhone and/or Apple Watch), and the Google Fit app. At the start of the two tracking weeks, participants were given specific instructions to wear their own activity tracker for 14 consecutive days and during 24 h a day or to carry their smartphone as frequently as possible to those using only the Google Fit app. Participants had access to different tutorials explaining the steps to follow to extract their device-based personal data (Fitbit, Apple, and Garmin brands) recorded over the 2 weeks and to upload them into their account on the study website. The Google Fit app gives the possibility to the user to blend data from multiple health apps and devices. Connection to an application programming interface was used to allow access and extraction of the data of the participants who agreed to share their personal data collected through the Google Fit app.

The most frequently used and relevant metric related to physical behaviors that could be obtained from the raw data of the different sources mentioned above was the number of steps accumulated per day, hereon referred to as daily step count. It is considered as a physical activity parameter that reflects the user's physical activity behavior and, especially, bipedal locomotion. This metric is considered to be a reliable proxy of the overall volume of the physical activity performed by individuals.^28^ A valid day with daily step count data was defined as a day characterized by an accumulation of at least 500 steps/day.^29,30^ Days with steps/day value <500 steps/day were thus removed from the analysis.

Personal data extracted and shared by FAY participants who used a Garmin watch during the 2-week tracking period did not include information on the number of steps accumulated during the day; therefore, Garmin watch users among the FAY participants were not included in the analysis of this study. We considered three categories for the daily step count measurement method (Fitbit watch users, Apple devices users, or Google Fit app users).

### Glucose monitoring and glycemic indices

The FreeStyle Libre flash glucose monitor (Freestyle Libre; Abbott Laboratories, Abbott Park, IL) was used to continuously measure glucose concentrations in the interstitial fluid during the 2-week tracking period of the FAY study. This glucose monitor includes two separate components. First, it requires the attachment of a small sensor to the upper arm via an adhesive patch, and secondly, a handheld device is also needed to read and download data from the sensor via near field communication. The sensor measures automatically, over 14 days and without need of calibration, interstitial glucose concentrations every 15 min (96 measures per day), and the handheld device displays and saves the data when the user scans the sensor. The sensor must be scanned at least once every 8 h to avoid data loss. Therefore, in this study, participants were asked to scan the sensor at least once every 8 h, and were encouraged to scan before going to sleep and in the morning upon waking.

Values of glucose concentrations were used to derive intraday glycemic control and variability indices for each day of the 2-week tracking period. Four measures of glycemic control were used within this study: minimum and maximum of daily glucose values, mean daily glucose, and the glucose management indicator that was computed using the equation 3.31 + (0.02392 × mean daily glucose in mg/dL).^31,32^ Three measures of glycemic variability were explored within this study: within day SD and within day CV of daily glucose values, and the approximate area under the sensor glucose curve over 24 h. These eight indices were obtained using the “cgmvariables” function of the “cgmanalysis” R package.^31^ The “cleandata” function of the “cgmanalysis” R package was also used to fill in gaps in glucose raw data <20 min using linear interpolation. Glycemic indices were derived using all glucose measures available with gaps <20 min imputed.

Glycemic control and variability indices of the first and last days of the 2-week tracking period were removed and not included in the analysis as they were partial days in terms of wearing the glucose monitor. For each participant, up to 13 complete days with glycemic indices were thus available.

### Covariates

All covariates were obtained from the baseline online questionnaires to which the FAY study participants had to answer before the 2-week tracking period. Self-reported height and weight at baseline were used to calculate body mass index (BMI). Smoking status was categorized as never, former, or current smoker. This last category included occasional and daily smokers. We also considered three categories for the self-declared stress level (low, medium, or high).

### Statistical analyses

Both glucose raw data (FreeStyle Libre output) and device-based physical activity raw data included the date of each measure. This allowed to synchronize retrospectively the data of the different devices and to match for each day of the two tracking weeks, daily glycemic indices to their corresponding physical activity measure. Only participants with at least 7 days, with both daily step count and glycemic control and variability indices available, were considered for the analysis.

Baseline characteristics of the study population were reported as means (SD) for continuous variables, median (interquartile range [IQR]) for skewed distributions, and as numbers (percentages) for categorical variables. A four-category physical activity classification based on Tudor-Locke's classification^28^ was used only for the description of the participants' characteristics. This four-category physical activity classification was based on daily step count averaged over the 13 tracking days and consisted of the following categories: sedentary (<5000 steps/day), low active (5000–7499), somewhat active (7500–9999), and active (≥10,000).

Generalized estimating equation (GEE) models were used to estimate associations of daily step count with glycemic control and variability indices. To assess the timing of potential associations between physical activity and glycemic control and variability, continuous daily step count (per 1000 steps/day) was studied in relationship to (1) glycemic control and variability indices of the same day, (2) glycemic control and variability indices of the next day, and (3) glycemic control and variability indices of the day after the next day.

The advantages of the GEE models are that they consider the within-individual correlation in exposure and outcome due to repeated measures. They can also better handle unbalanced data as the number of repeated measures per subject can be different from one participant to another. For each glycemic control and variability indices (outcomes), the three following correlation structures were tested and compared with one another using the quasi-likelihood under independence model criterion (QIC) value: unstructured (all correlations between observations are estimated separately), autoregressive (assumes a stronger correlation between two observations taken closer together in time and a decreasing correlation for farther time periods), and exchangeable (assumes one same correlation across all observations independently of the time periods between observations).^33^ For each glycemic index, the correlation structure associated with the lowest QIC value was selected and used for all analyses.

Models were calculated univariably and adjusted for age, sex, BMI, smoking status (never, former, or current smoker), self-declared stress level (low, medium, or high), and for the daily step count measurement method (Fitbit watch users, Apple devices users, or Google Fit app users). Sensitivity analyses were performed taking into account only days with at least 90% (86/96) of daily glucose values available in the raw data.

Statistical analyses were performed using the Statistical Analysis System software, version 9.4 (SAS Institute, Cary, NC), and two-sided *P*-values <0.05 were considered statistically significant. GEE models were performed using the PROC GENMOD procedure in the SAS software. Graphs were generated using the Matplotlib library in Python (version 3.8) software.

## Results

### Participant characteristics

**FIG. 1.**
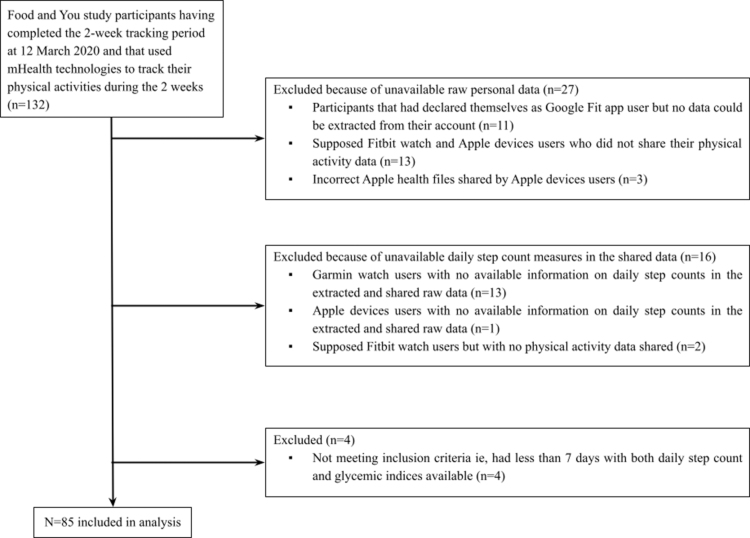
Flowchart of the study.

In March 2020, 132 FAY study participants had completed the 2-week tracking period and had, in principle, used mHealth technologies to track their physical activities during these 2 weeks. From these 132 participants, 47 were excluded either because of the total absence of any raw personal data (useable or shared) collected from mHealth technologies (*n* = 27), because of the absence of shared raw personal data related to daily step count (*n* = 16), or because of a number of days with both daily step count and glycemic indices available less than 7 (*n* = 4), leaving 85 participants in the study analysis (flow chart in Fig. 1).

Characteristics of the study population are displayed in Table 1. Daily step count averaged over the tracking days for each participant had a mean value in our study population of 8089.2 (SD 3479.5) steps/day and ranged from 2086 to 20,473 steps/day. The number of valid days, with both daily step count values and glycemic control and variability indices, ranged from 7 to 13 days, and its median was 13.0 (IQR: 1.0). Study participants used almost equally Apple devices (33/85, 39%) and the Google Fit app (30/85, 35%) to track their physical behaviors, whereas fewer participants used Fitbit watches (22/85, 26%). Participants were aged 40.1 years (SD 12.7) on average. Individuals in the higher step-defined physical activity categories seemed to be older than those in the lower categories. The study sample was composed of slightly more women (48/85, 57%). Participants had a mean BMI of 23.8 kg/m^2^ (SD 3.3) with 33% (28/85) overweight or obese individuals, and predominantly self-reported themselves as never or former smokers (71/85, 84%).

**Table 1.** Characteristics of the Study Sample (*n* = 85)

Mean (SD) or* n *(%)	All (*n* = 85)	Four-category step-defined physical activity classification				*P*
		Sedentary <5000 steps/day (*n* = 13)	Low active (5000–7500) (*n* = 30)	Somewhat active (7500–10,000) (*n* = 24)	Active ≥10,000 (*n* = 18)	
Daily step count (steps/day)^a^	8089.2 (3479.5)	3663.9 (858.4)	6255.8 (797.2)	8804.8 (718.8)	13,386.8 (2484.9)	<0.0001
Number of valid recording days^b^	13.0 (1.0)	13.0 (1.0)	13.0 (1.0)	13.0 (1.0)	13.0 (1.0)	
Daily step count measurement method						0.006
Fitbit watches	22 (25.9)	0 (0.0)	4 (13.3)	11 (45.9)	7 (38.9)	
Apple devices	33 (38.8)	5 (38.5)	12 (40.0)	8 (33.4)	8 (44.4)	
Google fit app	30 (35.3)	8 (61.5)	14 (46.7)	5 (20.9)	3 (16.7)	
Age, years	40.1 (12.7)	38.6 (15.6)	36.8 (8.8)	41.9 (13.0)	44.0 (15.0)	0.45
Sex						0.30
Male	37 (43.5)	4 (30.8)	17 (56.7)	10 (41.7)	6 (33.3)	
Female	48 (56.5)	9 (69.2)	13 (43.3)	14 (58.3)	12 (66.7)	
BMI, kg/m^2^	23.8 (3.3)	23.4 (3.2)	24.2 (3.3)	24.2 (3.9)	22.6 (1.9)	0.31
Categories of BMI						0.06
Normal weight, <25 kg/m^2^	57 (67.1)	10 (76.9)	16 (53.3)	15 (62.5)	16 (88.9)	
Overweight or obese, ≥25 kg/m^2^	28 (32.9)	3 (23.1)	14 (46.7)	9 (37.5)	2 (11.1)	
Waist circumference, cm	83.2 (12.1)	80.1 (8.9)	84.8 (12.6)	85.3 (14.3)	80.2 (9.8)	0.53
Hip circumference, cm	98.2 (8.2)	99.4 (10.4)	98.0 (7.8)	99.5 (9.0)	96.1 (5.9)	0.61
Work type related to physical activity						0.04
Sitting	63 (74.1)	11 (84.6)	26 (86.7)	17 (70.8)	9 (50.0)	
Doing light or hard physical work	22 (25.9)	2 (15.4)	4 (13.3)	7 (29.2)	9 (50.0)	
Self-declared stress level						0.26
High	13 (15.3)	1 (7.6)	4 (13.4)	4 (16.7)	4 (22.2)	
Medium	40 (47.1)	6 (46.2)	10 (33.4)	14 (58.3)	10 (55.6)	
Low	32 (37.6)	6 (46.2)	16 (53.4)	6 (25.0)	4 (22.2)	
Smoking status						0.63
Never	40 (47.1)	5 (38.5)	15 (50.0)	11 (45.8)	9 (50.0)	
Former	31 (36.4)	4 (30.7)	9 (30.0)	10 (41.7)	8 (44.4)	
Current	14 (16.4)	4 (30.7)	6 (20.0)	3 (12.5)	1 (5.6)	
Glycemic control indices^c^						
Minimum of glucose values, mg/dL	52.6 (11.3)	51.2 (8.2)	52.4 (10.5)	54.0 (14.5)	51.9 (10.3)	0.99
Maximum of glucose values, mg/dL	180.5 (28.9)	177.8 (26.1)	183.8 (31.8)	183.6 (32.8)	173.0 (19.1)	0.68
Mean glucose, mg/dL	93.6 (8.7)	90.4 (8.1)	95.9 (7.3)	94.2 (11.8)	91.4 (5.5)	0.10
Glucose management indicator, %	5.5 (0.2)	5.5 (0.2)	5.6 (0.2)	5.6 (0.3)	5.5 (0.1)	0.10
Minutes spent below 70 mg/dL, %^b^	3.0 (5.2)	4.9 (7.2)	1.7 (2.9)	3.8 (8.8)	2.0 (4.9)	
Minutes spent above 180 mg/dL, %^b^	0.08 (0.27)	0.17 (0.32)	0.16 (0.32)	0.04 (0.16)	0.00 (0.08)	
Minutes spent in the range 70–180 mg/dL, %^b^	97.0 (4.7)	94.8 (7.6)	97.9 (3.2)	96.0 (8.1)	97.9 (5.0)	
Glycemic variability indices^c^						
SD of glucose, mg/dL	16.8 (3.2)	17.0 (3.4)	17.0 (3.6)	16.8 (3.3)	16.2 (2.5)	0.84
CV of glucose, %	18.0 (3.3)	18.8 (3.3)	17.7 (3.5)	18.0 (3.6)	17.7 (2.5)	0.64
Approximate area under the sensor glucose curve, mg.h/Dl	14,989.6 (13,659.8)	12,068.8 (13,116.7)	15,886.8 (13,912.5)	18,364.6 (13,356.8)	11,103.7 (13,746.1)	0.20

*n* (%) and *P*-value of Fisher's exact test for categorical variables.

Mean (SD) and Kruskal–Wallis test for continuous variables.

^a^ Mean of daily step count computed taking into account valid days only.

^b^ Median (IQR) are presented instead of mean (SD) due to skewed distributions.

^c^ Evaluated from 2 weeks of Freestyle Libre-based CGM, excluding the first and last day of the tracking period.

BMI, body mass index; CGM, continuous glucose monitoring; CV, coefficient of variation; IQR, interquartile range; SD, standard deviation.

**FIG. 2.**
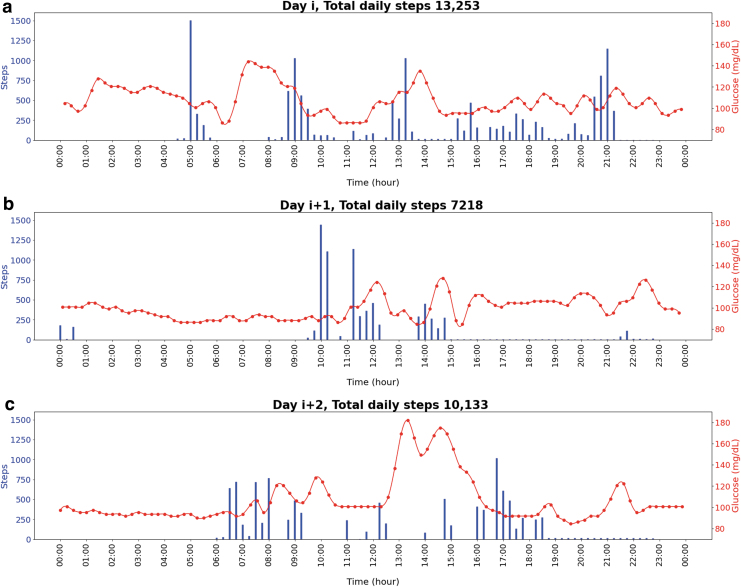
Evolution of the number of steps taken and interstitial glucose levels during day *i*, day *i* + 1, and day *i* + 2 for one study participant that had a mean daily step count of 6428 steps/day. Day *i* represents a random day during the 2-week tracking period. The numbers of steps cumulated were summed over 15 min epochs.

Figure 2 shows real-world examples of physical activity and glucose monitoring within 3 days of the 2-week tracking period for one FAY study participant.

### Associations between daily step count and glycemic indices

Associations between daily step count and glycemic control and variability indices using GEE analysis are presented in Table 2. In the multivariable analyses, no association was found between daily step count and glycemic control and variability indices of the same day.

**Table 2.** Associations Between Daily Step Count and Glycemic Control and Variability Indices (*n* = 85)

	Daily step count (1000 steps/day) same day^a^		Daily step count (1000 steps/day) next day^a^		Daily step count (1000 steps/day) 2 days after^a^	
	β (95% CI)	*P*	β (95% CI)	*P*	β (95% CI)	*P*
Glycemic control indices^b^						
Minimum of glucose values, mg/dL	0.0158 (−0.1045 to 0.1361)	0.80	−0.0470 (−0.1623 to 0.0683)	0.42	0.0542 (−0.0645 to 0.1729)	0.37
Maximum of glucose values, mg/dL	−0.0759 (−0.3758 to 0.2239)	0.62	**−0.3588 (−0.6931 to −0.0245)**	**0.035**	−0.1955 (−0.5170 to 0.1260)	0.23
Mean glucose, mg/dL	0.0069 (−0.0877 to 0.1014)	0.89	**−0.0917 (−0.1793 to −0.0042)**	**0.04**	0.0097 (−0.0828 to 0.1021)	0.84
Glucose management indicator, %	0.0002 (−0.0021 to 0.0024)	0.89	**−0.0022 (−0.0043 to −0.0001)**	**0.04**	0.0002 (−0.0020 to 0.0024)	0.84
Glycemic variability indices^b^						
SD of glucose, mg/dL	−0.0380 (−0.0944 to 0.0184)	0.19	−0.0365 (−0.1000 to 0.0270)	0.26	**−0.0623 (−0.1222 to −0.0023)**	**0.04**
CV of glucose, %	−0.0365 (−0.0932 to 0.0202)	0.21	−0.0221 (−0.0878 to 0.0436)	0.51	−0.0569 (−0.1152 to 0.0015)	0.056
Approximate area under the sensor glucose curve, mg.h/dL	10.9422 (−3.2709 to 25.1553)	0.13	2.1925 (−10.5745 to 14.9594)	0.74	10.1377 (−5.0377 to 25.3131)	0.19

Models adjusted for age, sex, BMI, smoking status, self-declared stress level, and for the daily step count measurement method.

Bold text indicates *P*-value ≤0.05.

^a^ Daily step count (steps/day) was studied in relation to glycemic control and variability indices of the same day, the next day and in relation to those of the day after the next day.

^b^ Evaluated from 2 weeks of Freestyle Libre-based CGM, excluding the first and last day of the tracking period.

CI, confidence interval.

However, when considering the glycemic control and variability indices of the next day, every 1000 steps/day increase in daily step count was associated with a 0.3588 mg/dL decrease (95% confidence interval [CI]: −0.6931 to −0.0245) in the maximum of glucose values, a 0.0917 mg/dL decrease (95% CI: −0.1793 to −0.0042) in the mean glucose, and a 0.0022% decrease (95% CI: −0.0043 to −0.0001) in the glucose management indicator.

Regarding glycemic control and variability indices of the day after the next day, we did not observe any association with daily step count except for the SD of glucose. Every 1000 steps/day increase in daily step count was associated with a 0.0623 mg/dL decrease (95% CI: −0.1222 to −0.0023) in SD of glucose 2 days later.

Results of the sensitivity analyses were substantially the same as those presented in Table 2 (data not tabulated).

## Discussion

### Principal findings and comparison with literature

Using data collected from mHealth technologies such as CGM devices, smartphone health apps, and consumer-based wearable activity trackers, our results suggest that daily step count was significantly and inversely associated with the maximum of glucose values, mean glucose, and the glucose management indicator of the next day. We also found that daily step count was significantly and inversely associated with SD of glucose of the day after the next day. This study did not reveal any association between daily step count and glycemic indices of the same day.

In the present study, no association was found between daily step count and glycemic indices of the same day. A similar study, conducted among healthy adults, investigated the relationships between physical behaviors assessed by accelerometers and CGM-based glycemic indices of the same day.^34^ They did not find any association between minutes spent in light physical activities, in moderate to vigorous physical activities, and mean and SD of glucose. However, when focusing only on lower fitness individuals, they found that mean glucose was inversely associated with light physical activity and moderate to vigorous physical activity and that SD of glucose was inversely associated with light physical activity. We were not able to replicate these findings as the FAY study protocol did not include any physical fitness test.

We observed a relationship between daily step count and glycemic control of the next day, in particular with the maximum of glucose values, mean glucose, and with the glucose management indicator. For instance, every 1000 steps/day increase in daily step count was associated with a 0.3588 mg/dL decrease in the maximum of glucose values of the next day. Thus, increasing the total volume of physical activity may be linked to slightly blunted glycemic excursions during the next day, resulting in reduced maximum glucose values and mean glucose. These results have already been observed in populations with diabetes. Indeed, Munan et al. found in a recent meta-analysis that exercise reduces mean glucose within the 24 h after the exercise sessions in adults with type 2 diabetes.^35^ To our knowledge, our work is the first observational study to report these associations in adults from the general population and under free-living conditions.

Most previous studies that explored the associations between physical activity and glycemic indices among adults without diabetes focused mainly on the relationship between exercise timing and postprandial glucose excursions.^36–40^ Objectives and design of these studies are very different from our study which makes the intercomparison of results very difficult. One of the major differences is that these studies were undertaken under laboratory settings and focused on effects of structured exercise, whereas our study concentrated on the total volume of habitual physical activity in real-life settings. Moreover, results were not consistent across these studies. On the contrary, some have found that postprandial exercise could lower postprandial glucose excursions and that preprandial exercise would not be associated with postprandial glucose excursions.^36–38^ On the contrary, others have reported that preprandial exercise could effectively reduce postprandial glucose concentrations.^39,40^ Furthermore, most of the published studies did not analyze the effects of acute exercise interventions on glycemic measures of the following days even though it may be interesting to look deeper into it given that physical activity may enhance insulin sensitivity for up to 72 h.^41^ Further randomized controlled trials that would explore the prolonged effect of physical activity on CGM-based glycemic measures are warranted to replicate and/or to pursue our findings further.

We also found that daily step count was inversely associated with SD of glucose of the day after the next day. It is difficult to compare this result with those from other studies as no study with similar design was conducted so far. However, in a recent trial conducted in nontrained healthy individuals, Figueira et al. found that acute exercise sessions were associated with reduced SD of glucose during the 12–18 h after the exercise sessions in comparison with preexercise values.^42^ They did not observe any reductions of SD of glucose within the 12 h after the exercise sessions. Our result could suggest that positive benefits of physical activity could be visible beyond 18 h after an exercise session. We highly encourage trials to explore this hypothesis further by looking at the effects of physical activity beyond the 18 h after an exercise session or after a certain cumulative volume of physical activity.

### Strengths and limitations

This study has numerous strengths. It is the first of its kind to study daily step count in relationship to measures of glycemic control and variability in adults from the general population in real-life settings. There is a paucity of studies on this subject in the literature, largely due to the lack of data simultaneously collected by CGM systems and mHealth devices in individuals without diabetes. This study is further strengthened by the fact that we used device-based and continuous measurements for both the exposure and the outcomes. Indeed, physical activity was assessed via consumer-based wearable activity trackers used by study participants in free-living conditions and not through self-reported measures, which are known to be prone to social desirability and recall bias.^43^ Glycemic indices were evaluated using data collected from 14 days of wearing the FreeStyle Libre glucose monitor. These measurement methods gave us the possibility to investigate the dynamic and temporal associations of daily step count with glycemic control and variability indices of the same day, the next day, and those of the day after the next day. Moreover, the sample size of this study can also be considered as one of its strengths. A very limited number of similar studies were conducted on up to 29 individuals.^34,36,37,42,44^

This study also has limitations. First, information on food intake was not available, so we could not control for it in our analysis. Furthermore, glucose concentrations displayed by the CGM device were not blind. Therefore, participants could have modified their behaviors (e.g., reducing caloric intakes, increasing physical activity) as a consequence of their glycemic measures. Our findings should be interpreted cautiously given that dietary intake, a significant factor that affects blood glucose levels, was likely to have influenced the magnitude of the associations observed. We adjusted our analysis for many potential confounding factors such as age, gender, BMI, smoking status, and self-reported stress level, another factor known to impact glycemia.^45,46^

Information on device wear time was not available, so we were not able to control for it in the analysis as it is usually done when analyzing accelerometer-based physical activity data. However, we observed an overall high level of protocol compliance regarding the wearing of the CGM device and the use of mHealth technologies among our study population given that the median number of valid days, with both daily step count and glycemic indices data, was 13.0 (IQR: 1.0). In addition, we also had access to sleep data assessed by mHealth technologies for 87% of the present study sample, which suggests a high compliance particularly for the wearing of the consumer wearable devices. We concluded that our study participants wore their device most of the days of the 2-week tracking period.

Our study can be seen as heterogeneous in terms of the measurement methods used to evaluate physical activity, as study participants used different mHealth technologies to monitor their daily step count, and it is not known exactly how comparable the measurements obtained from the three methods are. But we controlled our analysis for the daily step count measurement method, and mHealth technologies used by study participants (Fitbit watches, Apple devices, Google Fit app) have all been shown to be accurate for measuring step count in laboratory and/or free-living conditions.^47–52^ Furthermore, the study population had a rather high mean daily step count value of 8089.2 (SD 3479.5) steps/day, which may limit the generalizability of the findings to the general population. But the mean daily step count ranged from 2086 to 20,473 steps/day, showing that we still had a large variability in average physical activity levels within our population.

Finally, intensities of the physical activities performed daily were not accessible. Nonetheless, daily step count is an increasingly widespread and intuitive metric that can be easily used as a target for health benefits and ensure reproducibility of research results. With the expansion of wearable activity monitors and smartphones onto the commercial market, the daily step counts metric has become largely accessible among the general population, which justifies its use and emphasizes the need to study it in relationship to health outcomes.^53^ Working with physical activity data based on the number of steps accumulated during the day implies also that we only have information on ambulatory physical activities.^54^ Thus, participating in nonambulatory physical activities such as swimming or cycling may not have been taken into account properly. However, it is known that ambulatory physical activities such as walking remain a central component of physical activity in the general population.^53^

### Implications and perspectives

We found that increasing the overall level of physical activity was associated with reduced glucose maximum peaks the following day. Our work may have important public health implications as elevated glucose excursion is a risk factor for cardiovascular diseases in populations with and without diabetes.^55–57^ Despite the well-recognized benefits of physical activity for health and the prevention of chronic diseases, more recent estimates of physical inactivity suggest that about 27.5% of adults are not meeting the 2010 World Health Organization (WHO) physical activity Guidelines.^58,59^ One of the possible reasons that could explain why individuals do not follow the guidelines would be that the reward (e.g., decreased chronic disease risk) associated with physical activity is not tangible and appears too far in the future.^34^ Thus, being able to display to individuals some short-term health benefits or positive physiological consequences of people's physical behaviors as the ones observed in this work can be of high interest for motivation and could serve as a leverage to meet the physical activity guidelines. Indeed, potential physiological consequences of certain movement behaviors (e.g., increasing daily step count) can be integrated into health-related apps and represented as personalized feedback. This may motivate more individuals to follow the public health guidelines and/or to engage in healthy movement behaviors and maintain them over time.

Our study gives some insights into the links between physical activity and glycemic indices in the general population. Similar works should be replicated among other populations, in particular among people with diabetes treated with insulin, to improve, for instance, machine learning algorithms of hybrid closed-loop insulin delivery systems. Indeed, to improve closed-loop algorithms performance, not only relationships between physical activity and CGM-based glycemic indices should be better understood among populations with diabetes, but also algorithms should integrate data collected by consumer-based wearable activity trackers.^60^ Hybrid closed-loop algorithms use the data from a CGM device to automatically adjust insulin delivery through an insulin pump without the user's intervention.^61^ However, these systems still require the users to record their carbohydrate intakes and intensity and duration of their planned physical activities.^62^

Data continuously and passively collected from consumer-based wearable activity monitors constitute a unique, a rich and a real-time source of information that can allow the improvement of closed-loop algorithms for better glucose management and to alleviate patient's mental burden.^60,61^ Indeed, these trackers give access in real-time and real-life to accurate and objective measurements related to physical activity (e.g., steps per minute, heart rate) and sleep times, thus enabling automated physical activity and sleep detections. Physical behavior-related metrics from wearable activity trackers could be incorporated into hybrid closed-loop as complementary parameters to automate and adjust in real-time insulin deliveries without patient intervention.

Therefore, we highly encourage the use of consumer-based wearable activity monitors by people with diabetes as they can provide valuable data to users, health care professionals, and researchers to better understand how physical activity affects blood glucose levels in real-life settings and to optimize diabetes management. Data collected by these tools could also be used in large epidemiological cohorts or surveillance studies to better monitor participants' physical activity and contribute to the deep digital phenotyping of populations in real-life settings.^63^

## Conclusions

In summary, our study revealed that daily step count was inversely associated with certain glycemic control indices in adults from the general population in real life, in particular with maximum glucose values, mean glucose, and with the glucose management indicator of the next day. Increasing the total volume of physical activity may be linked to slightly reduce maximum glucose excursions during the next day among individuals without diabetes. Further studies are warranted to elucidate the associations between physical behaviors and glycemic measures in both larger normoglycemic populations and populations with diabetes.
